# Emerging Frontiers in Clinical Mycology: Innovations, Insights, and Impacts

**DOI:** 10.1111/myc.70139

**Published:** 2026-01-21

**Authors:** A. Apostolopoulou, A. G. Stewart, H. Yoon, J. M. Steinbrink, D. Z. P. Friedman, L. Ostrosky‐Zeichner, I. S. Schwartz, the Mycoses Study Group Education and Research Consortium (MSGERC)

**Affiliations:** ^1^ Division of Infectious Diseases, Department of Medicine New York Presbyterian/Weill Cornell Medical Center New York New York USA; ^2^ Transplant and Immunocompromised Host Infectious Diseases, Infectious Diseases Division Massachusetts General Hospital Boston Massachusetts USA; ^3^ Harvard Medical School Boston Massachusetts USA; ^4^ Division of Infectious Diseases, Department of Medicine Albert Einstein College of Medicine and Montefiore Medical Center Bronx New York USA; ^5^ Division of Infectious Diseases, Department of Medicine Duke University School of Medicine Durham North Carolina USA; ^6^ Section of Infectious Diseases and Public Health, Department of Medicine University of Chicago Chicago Illinois USA; ^7^ Division of Infectious Diseases McGovern Medical School at UTHealth Houston Texas USA

**Keywords:** artificial intelligence, climate change, diagnostics, fungal infection, mycoses

## Abstract

The Mycoses Study Group Education and Research Consortium (MSGERC)—a group comprising clinicians, researchers, patients, and industry partners—meets every 2 years to review the most significant challenges facing the clinical mycology community and plan research, education, and advocacy strategies to prevent fungal infections and improve outcomes. Key themes of the 2024 biennial meeting included emergence of antifungal resistance, the effects of climate change on incidence of IFI, recent healthcare‐and community‐associated outbreaks and their management, and the potential benefits of novel approaches, such as innovative study designs, host‐directed diagnostics and therapies, and artificial intelligence in clinical and academic mycology.

## 1 Introduction

The landscape of clinical mycology is changing, with a growing population of patients at heightened risk for invasive fungal infections (IFIs) due to immunosuppression, climate change, and community and healthcare‐associated outbreaks. At the same time, there have been promising advances in approaches to fungal disease surveillance, new diagnostics and novel treatments, and opportunities abound to advance the field using new ways of leveraging data.

The Mycoses Study Group Education and Research Consortium (MSGERC) is a non‐profit organization dedicated to promoting research, scholarship, education, and advocacy for the diagnosis and management of IFIs. Clinical Mycology Today, MSGERC's biennial meeting, was held September 4–6, 2024, in Colorado Springs, Colorado, and convened an international community of clinicians, researchers, educators, advocates, and patient and industry representatives. This past year's meeting addressed new and longstanding challenges in clinical mycology, including the use of artificial intelligence (AI) in research and clinical practice, enhancing public and governmental awareness of fungal pathogens and epidemics, and assessing the impact of climate change on the global incidence of fungal infections.

This review summarises the meeting's key topics and conversations and proposes a call‐to‐action to address challenges in clinical mycology.

## 2 Urgent Issues in Mycology

### 2.1 Fungal Adaptation to Extreme Conditions: Climate Change and Beyond

Climate change may result in altered patterns of fungal infections. Many fungal pathogens have environmental reservoirs, and changes to the environment may affect the likelihood of infections, pathogen virulence, and antifungal resistance.

Coccidioidomycosis is a major public health concern, with > 20,000 cases reported annually, which likely represent a fraction of the true burden of disease. There is accumulating evidence that climate change is leading to an increased geographic range and incidence of coccidioidomycosis. Wetter winters alternating with hotter and drier summers (a phenomenon called ‘hydroclimatic swings’) create conditions that favour *Coccidioides* hyphal growth, followed by desiccation, fragmentation, and aerosolization of arthroconidia. This so‐called ‘grow and blow’ hypothesis for *Coccidioides* growth and transmission is supported by an epidemiologic surveillance study in California (2000–2020) that demonstrated an association between such extreme weather conditions and an increased incidence of coccidioidomycosis [1]. Epidemiologists are forecasting a continued increase in cases of coccidioidomycosis in the arid regions of the Southwestern United States [2] (exacerbated by migration patterns, as rising costs lead to an influx of migrants in areas of higher risk, like central California) in addition to expansion of *Coccidioides* outside of areas of classic geographic risk [3, 4]. Climate change‐associated water scarcity is also responsible for an increase in wildland fires observed in western parts of the US, and these events can also increase the risk of coccidioidomycosis [5]. Outbreaks of coccidioidomycosis among wildland firefighters suggest an occupational risk for the disease in areas of geographic risk [6].

In other parts of the United States as well as many global regions, increased precipitation, rising sea levels, and increased frequency and severity of tropical storms converge to increase risks of flooding. Subsequent water damage to indoor structures results in increased exposure to moulds. Such exposure can lead to invasive mould infections in immunocompromised individuals. For example, an increase in cases of invasive mould infections was observed in Houston after Hurricane Harvey [7].

It has been posited that climate change can result in increased fungal pathogenicity, but empirical data are scarce. Fungi are able to adapt to adverse environmental conditions, including oxidative stress, changes in temperature, pH, and ultraviolet (UV) light exposure. Unsurprisingly, climate change is facilitating the proliferation and migration of thermophilic fungi and enabling the growth of species with phenotypic plasticity [8]. Adaptation to heat stress, also known as ‘thermotolerance’, is mediated by alterations in the fungal cell wall, metabolism, and production of mycotoxins that promote fungal survival [9, 10, 11]. In addition, fungal melanin is a well‐established adaptive mechanism that promotes survival in adverse conditions, including UV radiation stress, oxidative stress, and environmental antifungal exposure [12, 13]. These adaptive responses may have important implications for host pathogenesis [14, 15].

### 2.2 The Mechanics of Antifungal Resistance, Tolerance and Heteroresistance

The emergence of antifungal resistance remains a pressing issue in mycology. In 2022, the World Health Organization (WHO) published a fungal priority pathogen list that highlighted several antifungal resistant threats, including multidrug‐resistant *Candida auris*, azole‐resistant *Aspergillus fumigatus* , and the increasingly azole‐resistant *C. parapsilosis* [16]. Antifungal resistance can develop from the selective pressure of antifungal agents within a host or in the environment. Key elements contributing to antifungal resistance and fungal tolerance within the host include prolonged exposure to antifungals, immune system deficiencies that hinder fungal clearance, unfavourable antifungal pharmacokinetics in deep‐seated infections, and infections associated with biofilms [17, 18, 19, 20, 21]. Additionally, cell wall remodelling can enhance the ability of fungi to evade the immune system, resulting in persistent infections [22]. On the other hand, widespread agricultural use of fungicides is linked to the emergence of drug‐resistant isolates, best shown in azole‐resistant *Aspergillus fumigatus* [ 23, 24]. Alarmingly, a recent study demonstrated the ability of an approved agricultural fungicide to select for *A. fumigatus* resistant to olorofim, a first‐in‐class orotomide antifungal not yet approved anywhere [25].

The rate of antifungal drug discovery and approval is inadequate to address the escalating threat of antifungal resistance. A major obstacle lies in the regulatory requirements for large‐scale phase 3 clinical trials, which often impose stringent inclusion criteria that complicate patient recruitment. Furthermore, key populations, such as children, pregnant individuals, and immunocompromised hosts, are frequently excluded from studies and are therefore excluded from drug approvals. In the absence of such approvals, securing insurance coverage or accessing medications through expanded or compassionate use programs becomes an often insurmountable hurdle. Apart from antifungal resistance, persistent or relapsing infections can develop due to either tolerance or heteroresistance within a fungal population. Tolerance describes drug‐susceptible isolates that can grow despite inhibitory drug concentrations, a phenomenon that has been extensively studied in *Candida* species. These tolerant subpopulations demonstrate a slower metabolic rate and low intracellular drug uptake [26, 27]. Conversely, heteroresistance refers to heterogeneity in antifungal susceptibility within an otherwise uniform pathogen population. Genetic mechanisms of azole heteroresistance in *Cryptococcus* and *Candida* species, which escape detection by conventional phenotypic susceptibility assays, may explain some treatment failures [28, 29, 30]. Drug exposures can also influence heteroresistance. For example, in a recent study of high‐risk stem cell transplant recipients, Zhai et al. demonstrated that the selective pressure of echinocandin prophylaxis led to clonal expansion of micafungin‐heteroresistant strains of *Candida parapsilosis* and breakthrough infections [31].

### 2.3 Fungal Outbreaks

Recently, the infectious disease and public health communities have encountered several fungal outbreaks in healthcare and community settings that highlight the importance of fungal pathogens in public health. Two distinct, nosocomial outbreaks of *Fusarium solani* meningitis were reported in non‐contiguous Mexican cities [32, 33]. The first occurred from September 2022 until July 2023 and involved predominantly female patients who received peri‐procedural epidural anaesthesia in four hospitals in Durango, Mexico. Over a thousand patients were exposed, of whom 80 developed fungal meningitis and 41 died [32]. Initially, the case fatality rate was as high as 81%, but it decreased after the identification of *F. solani* as the cause and the protocolization of diagnosis and early initiation of systemic antifungal therapy. The second outbreak (January–May 2023) had a similar iatrogenic mode of transmission and took place in Matamoros, Mexico. However, what started from two outpatient anaesthesia clinics evolved into a large multistate and multinational outbreak involving both Mexican and American citizens who had received care at these clinics [33].

Additionally, an important occupational outbreak occured involving blastomycosis among workers at a paper mill in Michigan in 2022–2023. Investigators identified 162 cases of blastomycosis in the outbreak, including 18 individuals who required hospitalisation and 1 death [34]. The outbreak was the largest of its kind in US history and required that paper production be idled for 3 weeks [34], costing up to 14.5 million dollars [35]. Despite extensive environmental sampling, the source of the outbreak was not identified [34].

Outbreaks of zoonotic sporotrichosis have increased in tropical and subtropical regions over the last decade [36, 37]. Brazil has been facing a large sporotrichosis outbreak since 1998, with human and cat cases reported in most states [37, 38]. A recent genomic epidemiology study on clinical human and animal isolates of *Sporothrix brasiliensis* collected in Brazil demonstrated large genetic diversity and cat‐to‐cat and cat‐to‐human transmission of multiple independent lineages across the country, likely related to the movement of animals [38].

### 2.4 Diagnostic Challenges in Clinical Mycology

Most IFIs present a significant diagnostic challenge to clinicians for several reasons. Firstly, their incidence is relatively low in the general population, and so recognition may be more challenging than for common diseases; moreover, their concentration at tertiary medical centres may make them even more difficult to recognize for clinicians outside these settings [39]. Secondly, clinical manifestations and radiographic findings can vary, often depending on the immunologic status of the host [40, 41, 42]. In addition, emerging cancer‐directed therapies are expanding the diversity of immunocompromised or immunomodulated hosts, necessitating clinicians with a specialised understanding of altered immune pathways and host–pathogen interaction. Thirdly, microbiologic diagnosis often requires specific mycological assays (e.g., fungal antigen testing), direct microscopy, molecular assays, and special immunohistochemical stains, and diagnostics may be unavailable or require sendout with long turnaround times [43]. For some fungal diseases, there is no standardised diagnostic algorithm and/or the diagnostic yield of the available methodologies is imperfect [44]. Consequently, a high index of clinical suspicion combined with highly trained microbiology laboratory staff is essential for making an accurate and timely diagnosis. Delayed diagnosis of IFIs can lead to increased morbidity and mortality.

### 2.5 Challenges in Generating Evidence in Clinical Mycology

Traditional mycology study designs and outcome measures are increasingly inadequate to address the diagnostic and therapeutic challenges faced by present‐day clinicians and patients [45]. These challenges question the suitability of conventional research approaches for fungal diseases, which occur with lower incidence rates and atypical clinical courses, particularly among immunocompromised individuals. Furthermore, at risk patient populations now extend beyond those with classic risk factors such as advanced HIV or transplantation and encompass even rarer groups of patients whose immune systems are modified by novel biologic therapies. This diverse epidemiology impedes the design of studies that rely on homogenous risk stratification.

Aligned with these challenges, many existing guidelines are outdated and/or based on expert opinion without randomised controlled trial (RCT) data. For instance, no new treatments for coccidioidomycosis have been approved in nearly four decades, and the lack of dedicated trials since 2007 is concerning [46]. Additionally, patient advocates highlight the dire consequences of overlooking fungal diseases. These issues were emphasised during the recent Mycology Advocacy, Research, and Education (MyCARE) initiative—a Pan‐fungal Disease Patient, Caregiver, and Clinician Summit in partnership with MSGERC [47]. The International Society for Human and Animal Mycology (ISHAM) has also established a patient representation and advocacy working group to gather patient feedback globally on research gaps in clinical mycology that require further investigation (https://www.isham.org/working‐groups/patient‐advocacy/).

## 3 Call‐to‐Action: Leveraging New Technologies and Tools

### 3.1 Re‐Evaluating Research Approaches in Medical Mycology

These challenges raise questions about the applicability of traditional RCT designs and conventional outcome measures for addressing urgent unmet needs and generating meaningful evidence. There is growing awareness among leaders and funding bodies of the need for alternative study designs, epidemiological tools, and patient‐reported outcome measures (PROM) that can yield relevant evidence for real‐world practice.

Several study designs and tools that progress beyond the status quo in generating much‐needed evidence:

1. Utilise alternative study designs beyond traditional RCTs, such as target trial emulation, and/or incorporate biostatistical tools, such as causal mediation analysis, to infer causality from observational data. Ensure careful consideration of bias and the rigorous identification, measurement, and control of confounders to maintain the validity of the findings.
2. Leverage large, accessible, real‐world databases to design rigorous observational studies that achieve both power and generalizability, such as the National COVID Cohort Collaborative (N3C) [48], Epic Cosmos package, Centers for Medicare and Medicaid Services (CMS) database, FungiScope, and International Paediatric Fungal Network (https://www.ipfn.org).
3. Conduct natural history studies on rare diseases or patient populations who do not meet clinical trial criteria, which are crucial for understanding emerging at‐risk hosts due to involvement of alternative immune pathways. These cohorts can serve as external controls in single‐arm clinical trials [45, 49].
4. Innovate case definitions and outcome measures, using composite outcome measures such as the Desirability of Outcome Ranking (DOOR) response criteria or win ratio, to enhance the relevance and sensitivity of outcome measures and to circumvent the need for large sample sizes. Current efforts are redefining clinical response criteria in mycology trials to generate quality evidence for efficacy and adverse effects that matter to patients, allowing partial credit based on clinical importance [50].
  Furthermore, the inclusion of modern diagnostic tools and algorithms is crucial for defining cases, as in the instance of the *Aspergillus* PCR's inclusion in the recent guideline for defining invasive aspergillosis (IA) in hosts not meeting the EORTC‐MSGERC criteria [51, 52, 53]. This is especially relevant for clinical trial enrollment or monitoring responses in diseases caused by rare and resistant pathogens diagnosed using new molecular tests and point‐of‐care diagnostics.
5. Integrate surrogate outcome measures, such as early fungicidal activity (EFA) in cryptococcal meningitis [54], and patient‐reported outcome measures (PROMs) that can serve as primary or secondary trial endpoints. Additionally, advancements in natural language processing and other AI tools can enhance clinical trial design, participant screening, recruitment, and conduct.

Some of these alternative study tools must be applied carefully to avoid potential biases inherent in observational studies and perform appropriate sensitivity analyses. Nonetheless, they provide an acceptable alternative to the status quo and are crucial for advancing evidence generation. Such efforts should be undertaken in a multi‐centre, collaborative approach to maximise efficiency, sample size, and available expertise within the global mycology community.

### 3.2 Raising Fungal Awareness

The growing threat of IFI demands boosted awareness and comprehension across clinical, laboratory, and community settings [55]. Raising awareness is challenged by underdiagnosis in the clinical setting, absence of surveillance systems, and unsatisfactory fungal diagnostics. A study in the United States surveyed a nationally representative sample of 4677 adults and determined that 68.9% had never heard of any of the following: aspergillosis, candidiasis, coccidioidomycosis (‘Valley fever’), cryptococcosis, blastomycosis, histoplasmosis [56]. Healthcare provider awareness is also substandard. These shortfalls stem from insufficient healthcare system education, provision, and infrastructure [57]. A cross‐sectional survey of healthcare providers conducted in Washington State identified that the majority never or rarely considered a diagnosis of coccidioidomycosis [58]. This highlights the need for interventions to improve awareness, education, and training in both community and healthcare settings.

Fungal infections do not typically require mandatory reporting, and public health agencies rely predominantly on the voluntary notification by individual healthcare providers. The development of national and global registries aims to better capture relevant data and trends of fungal disease burden. Examples include the Leading International Fungal Education (LIFE) initiative [59] and the SENTRY Antimicrobial Surveillance Program. Limitations in existing fungal infection registries include: (1) restriction of inclusion to specific patient populations (e.g., solid organ transplant recipients), (2) restriction to certain geographical areas, (3) reporting data on only one type of fungal disease (e.g., *Candida* fungemia), and/or (4) inclusion of microbiological data without clinical adjudication. Generation of uniform and accurate data is the first step in raising awareness. Additionally, campaigns, such as the Centers for Disease Control and Prevention's Fungal Disease Awareness Week, aim to further address inadequacies in medical mycology training among clinicians [60]. Computerised decision‐support systems and the integration of AI and natural language processing into electronic medical record platforms are important supports for clinicians to better diagnose and monitor fungal disease [61, 62]. For instance, the European Society of Medical Mycology (ECMM) has developed a scoring system (ECMM EQUAL score) and decision‐support tool to assist both with diagnosis, management, and outcome monitoring of the most prevalent mycoses (https://www.ecmm.info/equal‐scores/). Generating infectious disease diagnostic order sets, increasing the availability of point‐of‐care testing, and reflexive testing in the laboratory are additional strategies to facilitate diagnosis in the appropriate setting.

### 3.3 One Health Approach in Mycology

**FIGURE 1:**
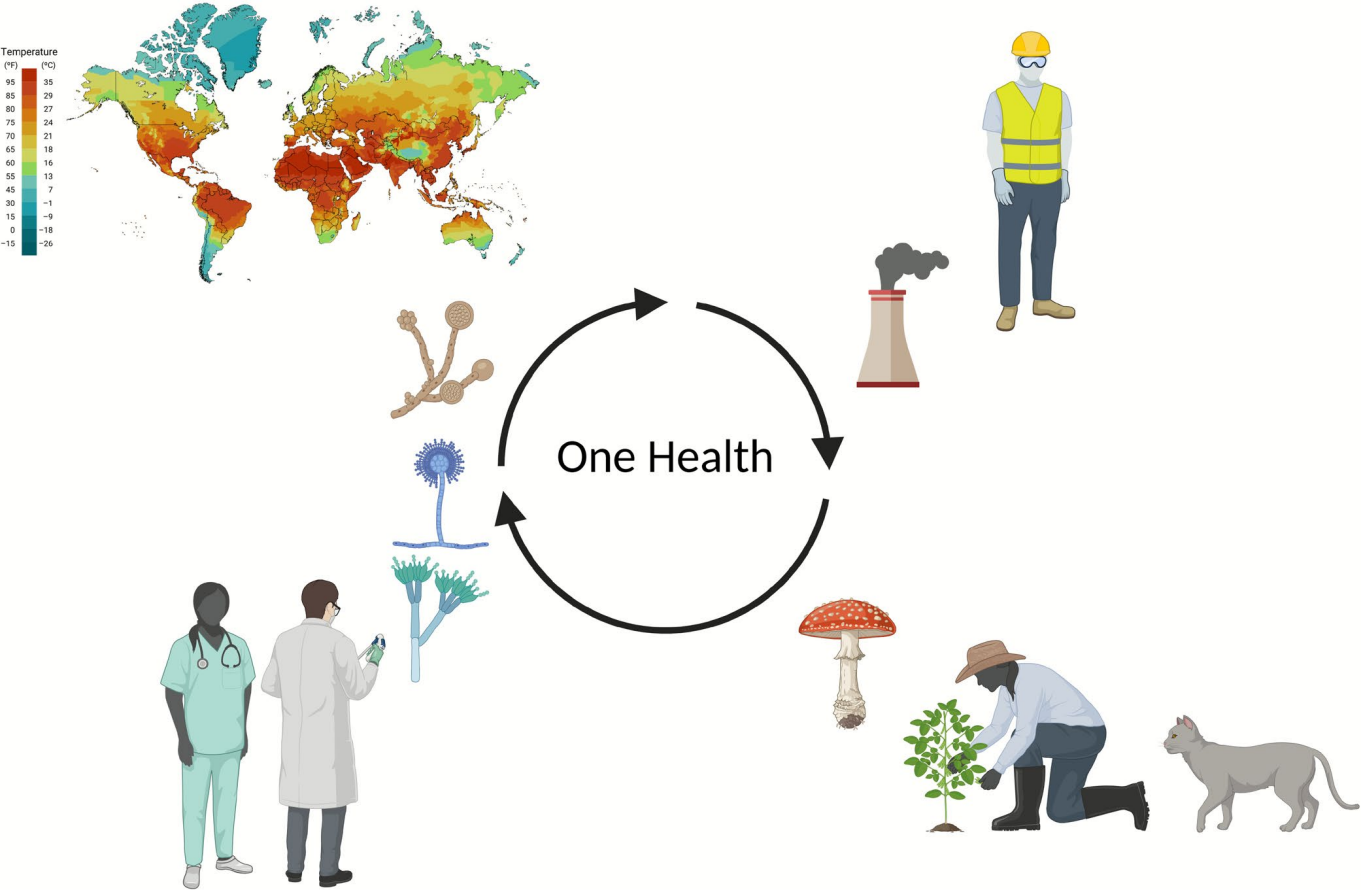
One Health approach requires collaboration between researchers, physicians, engineers, ecologists, plant health experts, and other disciplines to tackle the spread of mycoses.

A transdisciplinary One Health approach is required to tackle fungal disease. Fungal pathogens interact and spread between humans, animals, and the environment. Physicians and medical researchers must collaborate with veterinarians, environmental scientists, agricultural and plant health experts, ecologists and wildlife specialists, engineers and environmental designers, and behavioural scientists (Figure 1). Advocacy groups and non‐government organisations also play a crucial role in policy changes influencing the human‐animal‐environment interface. Without communication between these parties, widespread adverse outcomes and resource misallocation may ensue. Policy and behaviours that affect climate change are likely to contribute to outbreaks of fungal disease due to known and emerging pathogens [63]. Recognition of serious or atypical fungal infections by non‐ID medical specialties is pertinent to ensure favourable outcomes, as exemplified by the involvement of otolaryngologists in the diagnosis and management of rhinocerebral mucormycosis and dermatologists in the recognition of emerging *Trichophyton indotineae* dermatophytic outbreaks [64, 65].

### 3.4 To the Future: Precision Medicine and Artificial Intelligence

New technologies are transforming the way we approach IFIs. Precision healthcare has already made its impact in other fields of medicine with specific biomarkers to assess vulnerability to certain diseases, make diagnoses, or monitor response to treatment. Additionally, given significant heterogeneity within groups of patients, increasingly varied and personalised treatments are necessary. Therefore, not all patients at risk for or exposed to fungal pathogens develop invasive infections or respond to antifungals in the same way. These host differences are so distinct that available pathogen‐directed tests (i.e., galactomannan) perform differently across host risk groups. Therefore, efforts toward a more individual patient approach to fungal infection that considers host‐based factors, combined with pathogen‐based testing and clinical risk factors, are imperative [66, 67].

First, host‐based testing can determine patient‐specific biomarkers that identify susceptibility to fungal infection. These include individual gene mutations, whole blood gene expression changes, micro‐RNAs, and cytokines [66, 68, 69]. These markers can be helpful tools to develop personalised infection risk scores prior to immunosuppressive therapy to delineate who may benefit from antifungal prophylaxis compared to close monitoring alone.

Second, host‐based biomarkers can aid in diagnosing fungal infections. Current pathogen‐based fungal tests have many limitations, including shortcomings in accuracy and a delayed time to results. However, examining host immune responses, as measured by gene expression patterns in circulating leukocytes during infection, can provide an accurate, noninvasive method to diagnose fungal infection. Such unique and discriminatory host transcriptomic signatures can reliably detect fungal infection in both yeasts and moulds in in vitro, animal, and human studies [70, 71, 72]. When ultimately translated to a clinical platform, these tests will be able to accurately diagnose fungal infection across a wide array of hosts through a simple blood test.

Third, host‐based testing can be used for prognosis and to monitor response to treatment. A host biomarker can be baselined on an individual person either before an incident event (i.e., pre‐infection state) or at the time of diagnosis of fungal infection to trend biomarker responsiveness and monitor therapeutic effects. As an example, the host gene expression signature for candidemia showed a decrease in signal intensity over time with antifungal therapy, ultimately allowing the signature to predict the phenotype as healthy after antifungal treatment [72].

Potential therapeutic targets which augment the immune response to fungal pathogens have been identified. Both chimeric antigen receptor (CAR) T cells targeting *Aspergillus fumigatus* and immune checkpoint inhibitor therapy have demonstrated efficacy in preclinical mouse models [73, 74, 75, 76]. Lastly, pharmacogenomic approaches to improve azole antifungal drug exposure have also been advocated in the precision medicine approach [77].

**FIGURE 2:**
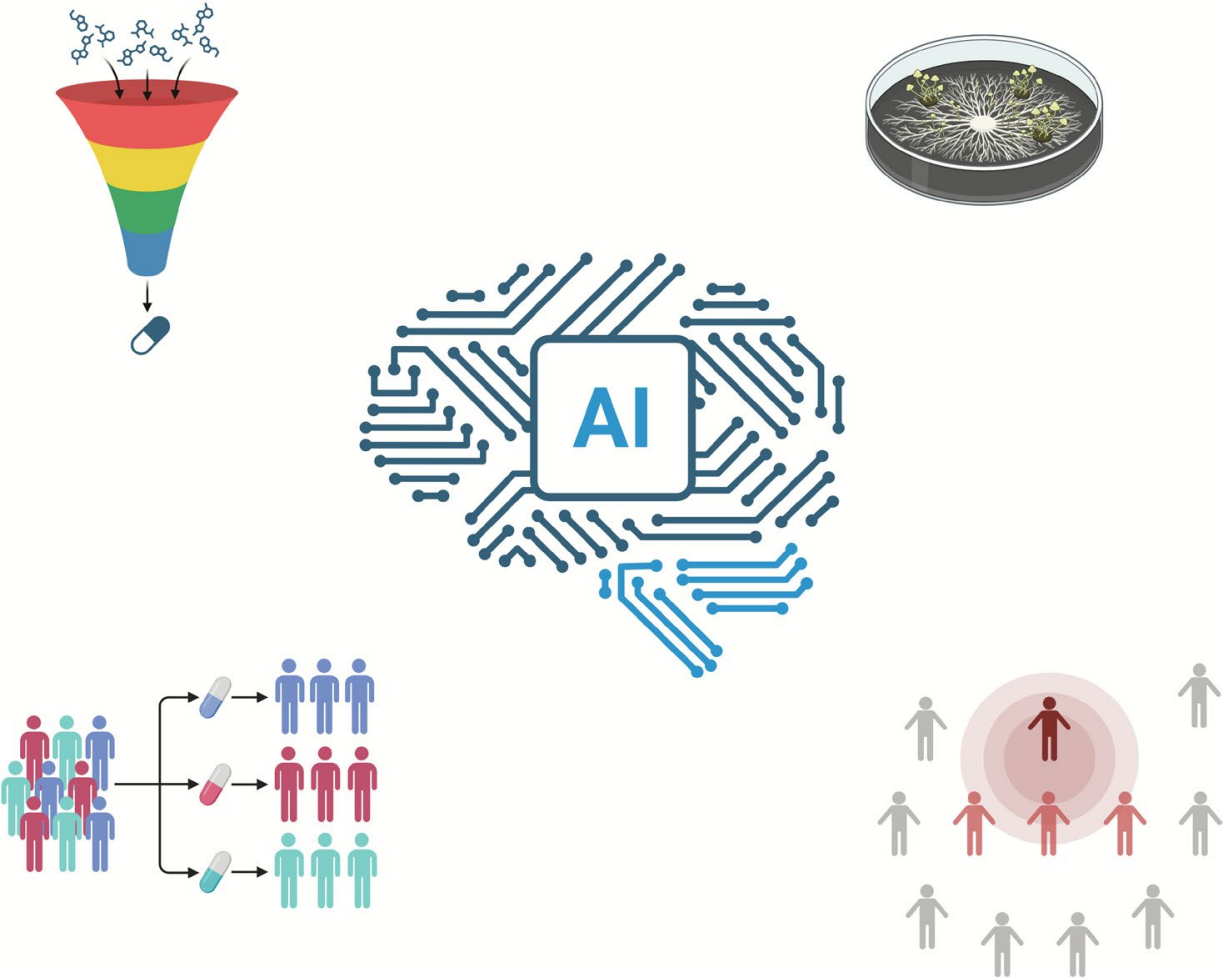
Potential applications of AI in Mycology may include compound screening for antifungal drug discovery (top left), clinical trial design and execution (bottom right), fungal identification (top right), and outbreak surveillance (bottom right).

AI can potentially improve several aspects of mycology, including automating outbreak detection, fungal identification, diagnosis, and—through discovery and clinical development of new therapies—the treatment of infections [78] as illustrated in Figure 2.

One area of promise for AI lies in the automation of outbreak detection. For example, Khanina et al. in Melbourne are developing an automated IFI surveillance tool that will integrate data derived from natural language processing from imaging, pathology, microbiology, and prescribing data among high‐risk inpatient populations to facilitate early detection [79].

AI tools may augment the diagnosis of mycoses. Machine vision (also called computer vision) is a type of AI system used for visual pattern recognition and could be leveraged for diagnostic applications in infectious diseases [80]. Examples of potential use cases being explored in mycology include the identification of clinical fungal isolates [81, 82, 83], the detection of and/or identification of fungi in histopathology or cytology slides [84], the identification of cutaneous lesions [85], the classification of fungal keratitis based on corneal imaging [78] and the differentiation of fungal lesions on radiographic studies [86].

Several groups have developed AI tools that can allow classification of fungi cultured from clinical specimens [81, 83, 86]. One group used a recurrent neural network algorithm to identify moulds. It was trained using nearly 100,000 images from 9 fungal taxa, and in testing it correctly classified moulds 90% of the time (compared to a gold standard of ITS sequencing and MALDI‐TOF), which was similar to specialist mould morphologists and superior to microbiology technicians, including those with additional training in mould identification [83]. Other investigators have used machine vision to identify yeasts based on colony morphology [82] and to differentiate hyphae of Mucorales from *Aspergillus* in tissue [84].

AI can accelerate the discovery and development of antifungal compounds, reducing time and cost [87]. Several groups have used AI to screen vast catalogues of purchasable compounds in the virtual space for those with properties of interest (like growth inhibition, lack of cytotoxicity, pharmacokinetic properties, etc.) [88]. To date, most high‐profile examples of AI‐facilitated anti‐infective molecule discovery have identified antibacterials, but this methodology can also be leveraged in antifungal development. One example is the use of machine learning to identify essentiality genes in *Candida albicans* , which may facilitate the identification of new antifungal targets [89]. A major technological advance that is now indispensable in rational drug design is AlphaFold 2, a transformer‐based model that allows prediction of three‐dimensional structures of proteins from amino acid or nucleotide sequences [90]. AlphaFold 3, the latest iteration, allows better prediction of protein–protein, protein‐ligand, and protein‐nucleic acid interactions [91].

AI may improve efficiency and reduce the costs of conducting clinical trials in mycology. One function is the use of digital twins—simulated controls that are generated by machine learning using prognostic models that incorporate a participant's baseline data with historical data about the natural history of a disease [92]. Digital twins might permit a reduction in the number of control‐arm participants. Another way AI can improve clinical trial efficiency and reduce costs is by automated screening and matching of participants to clinical trials. These approaches use large language models (LLM) to process unstructured text from notes in the electronic medical record to screen participants for clinical trial eligibility [93]. Potential cost savings can be substantial. For example, in a retrospective analysis, Unlu et al. used a retrieval augmented generation (RAG)‐enabled LLM to screen patient charts for inclusion in a heart failure trial and compared performance to human screeners. They found that the RAG‐LLM accuracy was similar to that of human screeners, but at a fraction of the cost [94].

AI tools are not without their limitations. Some challenges include the black‐box nature of many AI tools, limiting the ability of users to infer how a particular output was derived, which can limit trustworthiness. Moreover, AI tools can absorb and recapitulate biases present in their training data. In addition, generative AI tools can be limited by errors (colloquially called ‘hallucinations’ or ‘confabulations’), which currently preclude their safe use in most clinical contexts [95].

In conclusion, AI is a disruptive technology that can potentially improve several aspects of mycology, including clinical research, clinical diagnostics, and microbiology workflows, pre‐clinical drug discovery, and improved efficiency of clinical trials. Mycologists are encouraged to engage with AI tools to become familiar with their strengths and limitations.

## 4 Conclusion

The landscape of medical mycology is constantly evolving due to the expanding distribution of known pathogens, the emergence of new fungal species or resistance patterns, and the expansion and recognition of distinct patient populations at risk for infection. To adapt to this changing landscape, traditional research strategies and diagnostic tools must shift to more adaptive and relevant practices. Alternative study designs, consideration of host factors in diagnosis and management, the inclusion of AI, and refining diagnostic criteria and outcome measures are key tools to produce more meaningful and applicable patient‐centred evidence. To advance the field of mycology and surmount existing barriers, a coordinated global effort involving patients, clinicians, researchers, public health officials, and industry partners is imperative.

## Author Contributions

**A. Apostolopoulou:** conceptualization, methodology, writing – review and editing, writing – original draft, visualization, project administration. **A. G. Stewart:** conceptualization, methodology, writing – original draft, writing – review and editing, project administration. **H. Yoon:** conceptualization, methodology, writing – original draft, writing – review and editing. **J. M. Steinbrink:** conceptualization, methodology, writing – original draft, writing – review and editing. **D. Z. P. Friedman:** conceptualization, methodology, writing – original draft, writing – review and editing. **L. Ostrosky‐Zeichner:** writing – review and editing, supervision. **I. S. Schwartz:** conceptualization, methodology, writing – original draft, writing – review and editing, supervision, project administration.

## Funding

H.Y. was partially funded by the National Institute of Allergy and Infectious Diseases at the National Institutes of Health (K23‐AI177939) and the Irma L. and Abram S. Croll Charitable Trust. J.M.S. was partially funded by the National Institute of Allergy and Infectious Diseases at the National Institutes of Health (K23‐AI187748). L.O.‐Z. was partially funded by the National Center for Advancing Translational Sciences (NCATS), National Institutes of Health, through UTHealth‐CCTS grant number UL1TR003167 and contract U01CK000692, Centers for Disease Control and Prevention. The contents of this publication are solely the responsibility of the authors and do not necessarily represent the official views of the NIH or CDC.

## Conflicts of Interest

A.A., H.Y., and A.G.S. report no conflicts of interest. J.M.S. reports royalties from McGraw Hill, and is a co‐inventor on patents for gene expression classifiers of fungal infection, for which she receives royalty payments. D.Z.P.F. reports grants/research support from Cidara and Scynexis and speakers' honorarium from Eurofins. L.O.‐Z. has received research grants and/or consulting honoraria from the following companies: Scynexis, Melinta, GSK, Pulmocide, F2G, Basilea, Pfizer, Gilead, T2 biosystems, Octapharma, Meiji, Stendhal, Knight, and Eurofins Viracor. I.S.S. reports consulting honoraria from Pulmocide. 

## Data Availability

The authors have nothing to report.
